# Impacts of workplace verbal aggression classified via text mining on workers’ mental health

**DOI:** 10.1093/occmed/kqae009

**Published:** 2024-02-12

**Authors:** Y Nishimura, S Matsumoto, T Sasaki, T Kubo

**Affiliations:** Occupational Stress and Health Management Research Group, National Institute of Occupational Safety and Health, Kawasaki, Japan; Occupational Stress and Health Management Research Group, National Institute of Occupational Safety and Health, Kawasaki, Japan; Occupational Stress and Health Management Research Group, National Institute of Occupational Safety and Health, Kawasaki, Japan; Occupational Stress and Health Management Research Group, National Institute of Occupational Safety and Health, Kawasaki, Japan

## Abstract

**Background:**

Exposure to workplace aggression adversely affects workers’ health; however, little is known regarding the impact of specific types of verbal content.

**Aims:**

We aimed to examine the relationship between exposure to several types of aggressive words at work and the victim’s depressive symptoms and sleep disturbance using text mining.

**Methods:**

We conducted a longitudinal survey with 800 workers in wholesale and retail companies; of which, 500 responded to the follow-up survey. The Centre for Epidemiologic Studies—Depression Scale and Pittsburgh Sleep Quality Index were filled out by the participants, and their responses were analysed by logistic regression to evaluate the risk of depression or sleep problems. We collected exact aggressive words encountered at work over the past year as a dependent variable and classified it into four types using text mining, such as words criticizing one’s performance.

**Results:**

The follow-up rate was 63%. Exposure to words threatening one’s life showed a significant relationship with the risk of depression (odds ratio [OR] = 13.94, 95% confidence interval [CI] = 1.76–110.56). The exposure to words criticizing one’s job performance is significantly related to the risk of sleep disturbance (OR = 5.56, 95% CI = 2.08–14.88).

**Conclusions:**

These findings suggest that different contents of verbal aggression can have different impacts on workers’ health. This indicates that not only overtly threatening and abusive language but also words related to one’s performance can be a risk factor for workers, depending on how they are delivered. To mitigate the adverse effects, promoting effective communication and cultivating psychological detachment from work may be beneficial.

## Teaser

We explored the relationship of exposure to specific words of verbal aggression at work and depression and sleep. Our survey of 800 wholesale and retail workers, coupled with text mining, revealed that life-threatening language may escalate depression risk, whereas job-critical language may disrupt sleep. These findings emphasize diverse language effects on worker’s health, highlighting the need for improved workplace communication and psychological detachment from work.

**Key learning points**

**What is already known about this subject**

- Exposure to verbal aggression, such as workplace bullying, adversely affects workers’ mental health.
- However, it is currently unclear whether there are differences in the adverse effects of verbal aggression based on its content.

**What this study adds**

- The present study revealed that different types of aggressive words might affect workers’ mental health and sleep differently.
- Exposure to the life-threatening words relates to the risk of having depressive symptoms.
- Job-critical language affected sleep.

**What impact this may have on practice or policy**

- Given that our findings suggested the importance of assessing the content of the verbal aggression, objectively evaluating such content could enhance verbal abuse risk assessment in both practical and academic settings.

## Introduction

Verbal aggression in the workplace, such as workplace bullying, is one of many psychosocial job stress factors observed in the work environment [1]. Earlier studies, including our work based on the Japanese industrial accident compensation insurance records, have indicated that interpersonal conflict is a significant stressor in the workplace [2,3]. Customer and employee aggression can affect workers’ psychosocial environment, which may impair work performance and raise mental health issues [4,5]. Studies have reported that both depressive symptoms [6] and sleep problems were associated with adverse events at the workplace [7].

Verbal aggression is seriously problematic due to its wide reach, considering that the number of victims affected by verbal aggression far outnumbers those affected by physical aggression [8]. The Customer Harassment Survey 2022 conducted by the Japanese Trade Union Confederation revealed that 55% of those who have been victims of harassment in the last 3 years reported verbal abuse, which was significantly higher than the 7% who reported physical violence during the same period. Verbal aggression has a profound and widespread effect on not only the victim of the aggression but also other witnesses. A longitudinal study focused on the contextual effects of workplace bullying has reported increased psychological distress and intention to leave, even among workers not personally victimized [9]. A report from three experiments also shows that witnessing rudeness reduces the observer’s task performance in both routine and creative tasks [10]. Another study also reported that witnessing verbal aggression negatively affects witnessing customers, which may impair company sales [11].

As discussed earlier, previous studies have mainly focused on the prevalence and effects of workplace bullying and verbal aggression. However, little attention has been paid to the content of verbal aggression. For instance, a study that analysed language aggression in a virtual professional community reported two major forms of aggression and some sub-forms, such as dehumanizing and negative evaluations [12]; however, the authors did not investigate the health issues associated with the aggression. Employing the text mining technique, wherein Japanese sentences are separated into morphemes and morphologically analysed, allows for objective data-driven analysis of Japanese sentences [13]. Therefore, we collected verbally aggressive words which the participants were exposed to at their workplace, classified them using text mining, and then assessed their effect on workers’ depressive symptoms and sleep disturbances. Based on a previous study on online language aggression [12] and reports of customer harassment [8], we focus specifically on aggressive words regarding workability, attacks on personality that do not directly refer to work and threats on life.

The current study investigated sleep disturbance as an outcome given its importance in maintaining high productivity and good health among workers. Sleep, which is our primary source of recovery, has been associated with several health and productivity problems, such as stroke [14], depression [15] and cognitive abilities [16]. In particular, evidence suggests that sleep problems and depression are closely related [17], and being subjected to or witnessing bullying at work is associated with sleep difficulties [18]. Therefore, investigating the negative impact of verbal aggression, particularly the type of words used, on sleep quality may provide deeper insight into occupational health.

Herein, we aimed to investigate the effects of different types of aggressive words exposed at work on the worker’s depressive symptoms and sleep quality through a web-based longitudinal study. We recruited participants working in the wholesale and retail industries, given the high prevalence of vocal aggression victims in such industries based on the analysis of the statistics of the Industry Accident Compensation Insurance of Japan, which also compensates for work-related mental disorders. We hypothesized that exposure to life-threatening verbal abuse, such as ‘kill you’, and verbal aggression that criticizes one’s ability to work, such as ‘useless’ while at work, would have a profound impact on workers’ mental health.

## Methods

A longitudinal internet survey targeting Japanese workers in wholesale and retail companies was conducted in December 2020 and December 2021. The survey was conducted by a research company that randomly sends participation requests by e-mail to their participants’ pool consists of 1.12 million registrants. All participants received reward points worth 110 yen in accordance with the terms and conditions of the survey company for their participation. The initial sample size was determined considering the average follow-up rate (around 60% per year), average prevalence rates of the Centre for Epidemiologic Studies Scale (CES-D) and Pittsburgh Sleep Quality Index (PSQI) (20–30% and 30–40%) of previous surveys, including ours, and the suggested number of events per variable by Peduzzi *et al*. [19].

The questionnaire used in both surveys asked experience with hearing aggressive words targeted towards themselves and others at their workplace and the specific words of the verbal aggression within the past year using free-form questions. The exposure history collected at the second wave was used as an independent variable in the analysis. In addition, Japanese versions of the CES-D and PSQI were filled out to assess the risk of depression and sleep quality. As covariates, we also determined their demographic data, such as their biological sex, age, job classification, night shift and marital status.

All participants provided web-based informed consent prior to participation. The current study was approved by the Research Ethics Committee of the National Institute of Occupational Safety and Health, Japan (2020N14, 2021N25).

Exposure to aggressive workplace language within the past year, including those targeted to others, collected at the second survey was determined as an explanatory variable of depressive symptoms or sleep disturbances. A five-point Likert scale collected the exposure, comprising never, once or less in a month, a few times a month, one to few times a week and mostly every day. Thereafter, we dichotomized the response into ‘no exposure’ and ‘exposed’. The perpetrator of the aggression was also determined. Given the difficulty in determining aggressive and non-aggressive words using a unified criterion, we recorded any experience the participants felt as verbal aggression as a valid exposure. Aside from the experience of hearing aggressive words, we determined the words used for the verbal aggression that they experienced at their workplace through a free-form question.

The degree of depressive symptoms was assessed using the Japanese version of the CES-D [20]. The CES-D is a widely used and highly reliable screening tool for depression comprising 20 items questioning about physical and mental state over the past week from the day of the survey. Consistent with previous studies, we set the cut-off point at 16 points. The degree of sleep disturbances was assessed using the Japanese version of the PSQI [21]. The PSQI is a widely used, self-rated index that measures sleep quality and disturbances. It consists of 18 items questioning about normal sleep habits over the past month from the day of the survey. Again, consistent with previous studies, we set the cut-off point at 6 points. The results of CES-D and PSQI of the follow-up survey were used as outcomes, and the results of the baseline survey were used as covariates of the statistical analysis.

The demographic data collected consisted of biological sex (male or female), age (coded to every 10 years), job classification (5 types), night shift work (applicable or not) and marital status (married or not). These data were then used as covariates for subsequent analysis. Table 1 summarizes the demographic data of the participants. The proportion of changes in demographic information from waves 1 to 2 were as follows: job classification 10% (*n* = 51), night shift work 6% (*n* = 31) and marital status 2% (*n* = 11).

**Table 1.** Demographic data of the participants

	All	Not exposed	Exposed
Characteristic	*n*(%) = 500	*n*(%) = 392	*n*(%) = 108
Sex			
Male	296 (59)	238 (61)	58 (54)
Female	204 (41)	154 (39)	50 (46)
Age			
20s	20 (4)	16 (4)	4 (4)
30s	82 (16)	64 (16)	18 (17)
40s	143 (29)	102 (26)	41 (38)
50s	174 (35)	136 (35)	38 (35)
60–65	81 (16)	74 (19)	7 (7)
Mean (SD)	49.0 (9.9)	49.6 (10.1)	46.9 (9.1)
Marriage status			
Unmarried	202 (40)	156 (40)	46 (43)
Married	298 (60)	236 (60)	62 (57)
Job industry			
Wholesale and retail trade	500 (100)	392 (100)	108 (100)
Job class			
Regular employee	145 (29)	116 (30)	29 (27)
Managerial class	97 (19)	73 (19)	24 (22)
Board member	20 (4)	17 (4)	3 (3)
Non-regular employee	174 (35)	127 (32)	47 (44)
Self-employed	64 (13)	59 (15)	5 (5)
Night shift			
Applicable	38 (8)	30 (8)	8 (7)
Not applicable	462 (92)	362 (92)	100 (93)
The Centre for Epidemiologic Studies—Depression Scale			
Qualified (≥16)	120 (24)	80 (20)	40 (37)
Not qualified	380 (76)	312 (80)	68 (63)
Mean score (SD)	12.5 (7.9)	11.8 (7.2)	15.4 (9.5)
Pittsburgh Sleep Quality Index			
Qualified (≥6)	180 (36)	125 (32)	55 (51)
Not qualified	320 (64)	267 (68)	53 (49)
Mean score (SD)	5.1 (2.6)	4.8 (2.4)	6.2 (2.9)

All demographic data shown here were collected at the first survey.

We analysed the collected examples of aggressive words using the KH Coder with MeCab, a software for metric text analysis and text mining [22], which involved the following procedures: (A) separating sentences into morphemes, (B) summarizing word conjugations and (C) flagging the types of aggressive words. Flagging was conducted based on the inclusion of the following four types of aggressive words in each sentence: (i) criticizing one’s job performance (words directly related to one’s occupational life, e.g. idiot, useless, poor memory, quit and resign); (ii) attack on one’s personality and looks (words insulting one’s general personality and looks, e.g. bald head, shit, and ugly looking); (3) threats on life (e.g. die and kill) and (4) others (all examples not included in the aforementioned classifications). Given that the flags were set based on the presence of certain words in each collected example, there were cases in which one example corresponded to multiple flags. We determined the aforementioned classification based on a previous study on online language aggression [12] and the actual content of the collected examples. The regex terms used to detect each aggression type are detailed in Table 1 (available as Supplementary data at *Occupational Medicine* Online).

We tabulated the frequency of the extracted morphemes to illustrate the data and created word clouds for each aggressive word type using the R wordcloud2 package [23]. A word cloud is a visual representation of text mining results that displays words in varying sizes based on their frequency in the analysed text. Only words with a frequency of two or higher were included in the plot.

To determine the effects of verbal aggression during work, we conducted a multivariate logistic regression analysis, reporting the odds ratios (ORs) and 95% confidence intervals (95% CIs) for satisfying the criteria of depressive symptoms or poor sleep quality assessed by CES-D and PSQI, respectively. The adjusted model included sex, age class (every ten years), job class, night shift work and marital status. In the model using CES-D as an outcome, the baseline CES-D results were included as an additional adjustment factor. Similarly, in the model using PSQI as an outcome, the baseline PSQI results were included as an additional adjustment factor. The significance level of statistical tests was set at *P* = 0.05.

## Results

The demographic data of this study population are shown in Table 1. Of the 800 participants at the baseline survey, 500 of them also participated in the follow-up survey after one year (follow-up rate 63%). Among the 500 participants, 125 (25%) and 186 (37%) exceeded the threshold of CES-D and PSQI in the follow-up survey, respectively. Table 2 shows the number of participants exposed to verbal aggression at work according to each language type. With regard to the perpetrator of the aggression, victims most frequently reported aggression from their supervisor (Table 2, available as Supplementary data at *Occupational Medicine* Online).

**Table 2.** Number of participants exposed to each type of aggressive words

		CES-D^a^			PSQI^b^	
	Qualified (≥16)	Not qualified	Total	Qualified (≥6)	Not qualified	Total
Overall exposure	44	64	108	64	44	108
Exposure by type of aggressive words^c^						
Criticizing one’s job performance	20	17	37	27	10	37
Attack on one’s personality and looks	10	11	21	12	9	21
Threats on life	7	2	9	6	3	9
Other	24	48	72	42	30	72

*n* = 500;

^a^The Centre for Epidemiologic Studies—Depression Scale at follow-up.

^b^Pittsburgh Sleep Quality Index at follow-up.

^c^There were cases in which one example of aggressive words may be flagged with multiple types.

**Figure 1.**
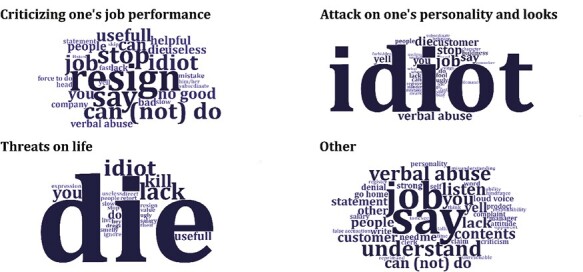
Word clouds for each type of aggressive words. Letter size represents the relative frequency of occurrence. In preparing the word frequency table, word conjugations were grouped together and then translated into English, so the plotted English words may differ from the examples cited in the paper. Only words with a frequency of two or higher are plotted.

Figure 1 shows the results of text mining the collected examples as word clouds. Below are some examples of the aggressive language collected during the current study (translated from Japanese to English by the authors). The translated English words in the word clouds may slightly differ from the examples cited below due to the timing of the translation from Japanese (before or after the morphological analysis).

‘Lose weight, fatty!’ ‘Fool, idiot!’ ‘Ugly, smelly, please die.’ ‘Don’t you understand Japanese? Are you mute or deaf?’ ‘Go home, you idiot!’ ‘Please die.’ ‘You idiot. Hey, I’ll kill you. Die, you son of a bitch!’ ‘Resign your job! I’ll tell the owner to make you quit!’ ‘Useless!’ ‘Deadhead, we don’t need you!’ ‘Resign by yourself. You’re an eyesore!’ ‘I hope you quit.’ ‘Don’t come to work anymore!’ ‘I know I shouldn’t discriminate, but you’re the one who didn’t have children, so you can’t think about others.’

Table 3 shows the ORs for satisfying the CES-D or PSQI criteria for being exposed to any of the verbal aggression (overall exposure) or each type of aggressive words. The comprehensive analysis showed that exposure to any type of the aggression relates to the risk of having depression and low sleep quality (OR = 1.97, 95% CI = 1.08–3.57 and OR = 2.91, 95% CI = 1.66–5.08, respectively). The exposure to the language type ‘threats on life’ showed a significant relationship with depressive symptoms (CES-D) in the adjusted model (OR = 13.94, 95% CI = 1.76–110.56). Although exposure to ‘Attack on one’s personality and looks’ and ‘Criticizing one’s job performance’ also negatively related with depressive symptoms regarding crude OR, the adjusted ORs did not reach significance. Our analysis regarding sleep problems found a significant relationship between a history of exposure to ‘criticizing one’s job performance’ and ‘other’, and the sleep quality (OR = 5.56, 95% CI = 2.08–14.88 and OR = 2.16, 95% CI = 1.12–4.17, respectively).

**Table 3.** Odds ratios determining the relationship between exposure to each type of aggressive words and outcomes

	Outcome = the CES-D^a^				Outcome = the PSQI^b^			
	Crude OR (95% CI)	*P*	Adjusted OR (95% CI)^c^	*P*	Crude OR (95% CI)	*P*	Adjusted OR (95% CI)^c^	*P*
Overall exposure	**2.64 (2.07, 5.00)**	**<0.001**	**1.97 (1.08, 3.57)**	**0.027**	**3.22 (2.07, 5.00)**	**<0.001**	**2.91 (1.66, 5.08)**	**<0.001**
By type of aggressive words								
Criticizing one’s job performance	**4.01 (2.03, 7.93)**	**<0.001**	2.01 (0.77, 5.26)	0.155	**5.16 (2.44, 10.93)**	**<0.001**	**5.56 (2.08, 14.88)**	**<0.001**
Attack on one’s personality and looks	**2.88 (1.19, 6.95)**	**0.019**	0.38 (0.1, 1.53)	0.169	2.34 (0.97, 5.66)	0.060	0.46 (0.13, 1.64)	0.235
Threats on life	**11.06 (2.27, 53.98)**	**0.003**	**13.94 (1.76, 110.56)**	**0.008**	3.46 (0.85, 13.99)	0.082	4.65 (0.72, 29.98)	0.107
Other	1.62 (0.94, 2.77)	0.08	1.6 (0.79, 3.24)	0.195	**2.76 (1.66, 4.6)**	**<0.001**	**2.16 (1.12, 4.17)**	**0.021**

*n* = 500.

^a^The Centre for Epidemiologic Studies—Depression Scale (CES-D).

^b^Pittsburgh Sleep Quality Index (PSQI).

^c^Adjusted for sex, age, marriage status, job class, night shift work, and the outcome measure at the baseline survey (CES-D or PSQI). Statistically significant results are shown in bold.

## Discussion

The present study aimed to investigate the effects of occupational exposure to aggressive words classified using text mining on depressive symptoms and sleep disturbances among wholesale and retail workers. Our findings showed that exposure to verbal aggression which threatens one’s life is associated with the risk of developing depressive symptoms evaluated using CES-D. Moreover, we found that exposure to criticism of one’s job performance relates to sleep quality evaluated using the PSQI. Although several studies have investigated the relationship between verbal aggression and mental problems, this study specifically focused on the content of aggressive words, which had not been investigated before.

Focusing on the effects of verbal aggression on depressive symptoms, we found a significant relationship with exposure to the ‘threats on life’. Exposures to aggression towards one’s personality and job performance also showed significant association before adjusting OR but did not reach significance after adjusting for covariates. Life-threatening event relates to depression [24]. Although the risk of being actually killed is thought to have been low, exposure to life-threatening verbal abuse may have triggered various stress responses, including hormonal and other endocrine responses. These responses might lead to depressive symptoms in the exposed workers through stress hormones and inflammations [25].

Focusing on the effects of verbal aggression on sleep disturbance, we found a significant relationship between exposure to words criticizing one’s job performance and the risk of exceeding the cut-off point for the PSQI. Exposure to other types of aggressive words showed no significant relationship with sleep in the adjusted model. While studies have already shown that workplace bullying affects the sleep of the victims and witnesses [18], our findings provided more detailed information on the relationship between workplace bullying and sleep disturbances. Although forceful criticism of one’s job performance is not uncommon in a work setting, persons initiating the criticisms may need to be mindful of how they are delivered to prevent sleep disturbances among recipients.

Our study found that different types of aggressive words mya have varying effects on mental problems, as shown by a significant effect of ‘threats on life’ on depression and ‘criticizing one’s job performance’ on sleep quality. Thus, while attacks on one’s existence and personality directly affect victims’ mental health, attacks on one’s job performance may mainly affect detachment from work. For example, those criticized for their job performance may have stayed longer at work to satisfy the requirements of the preparator, which could lead to insufficient recovery sleep due to shortened sleeping hours and interference with falling asleep [26,27]. Moreover, previous studies have shown an association between failure to detach from work psychologically and poor sleep [28]. Therefore, strong words regarding job performance may interfere with sleep by hindering psychological detachment. While preventing verbal abuse is of utmost importance, research on the role of psychological detachment from work could provide insights into reducing the harm caused by verbal abuse and aiding in recovery.

The current study has some strengths and limitations that need to be acknowledged. Our free-form questions and text mining allowed for a more profound analysis than previously conducted. Although the study was prospective; narrow range of examples and population should be improved in the feature. Fewer examples may also bias the classification of aggressive words. Since we recruited participants online by notifying the study’s primary purpose, the response rate could be higher among those who experienced the event. Thus, it should be noted that this may have biased the exposure estimates. Although the adjusted ORs for exposure to ‘an attack on one’s personality and looks’ did not reach statistical significance, they revealed an unexpected relationship. Although the crude ORs showed a hypothesized association, adding a baseline score of CES-D or PSQI as covariates reversed the effect of an ‘attack on one’s personality and looks’. This suggests that the impact of words that fall under this category varies widely, from positive to negative, depending on the victim’s mental state. A more extensive cohort study may be necessary to address this point. Considering the current study focused on workers in Japan’s wholesale and retail industries, our results might not be generalized to other cohorts. Finally, future studies must focus on broader work-related issues. Although sleep and depression are good starting points for assessing the psychosocial environment of one’s workplace, several other factors, especially work performance [29], should be investigated further.

This study suggested that verbal aggression widely relates with workers’ health, which is in agreement with previous findings [9–11]. Therefore, countermeasures to suppress verbal aggression at work should be prioritized more than ever. In the current study, participants identified their supervisors as the most frequent perpetrators of aggressive behaviour in the workplace. Previous research on destructive leadership underscores the need for managers to be educated on creating a healthy and respectful work environment where such behaviours are not tolerated [30]. Although prevention is crucial, it is equally important to provide adequate care to the victims for promoting a safe and healthy work environment. According to Andersen *et al*., social support, organizational justice and safety perceptions may help to reduce the risk of mental health issues for victims of workplace violence [31]. Moreover, improving one’s sleep has been shown to promote physical and mental recovery and reduce the risk of mental illness [32]. Hence, disseminating suggestions for improving sleep may be advantageous given its relatively lower cost and broader reach compared to medical interventions. Feature research should address above issues and also poor mental health of the perpetrator [33] to bring better work environment to life.

Given our findings suggesting the importance of not only the presence of verbal abuse in the workplace, but also the specific content of the aggression, an objective evaluation of such content could enhance verbal abuse risk assessment in both practical and academic settings, assuming further study development. In addition, employing psychophysiological approaches, such as event-related potentials on electroencephalography or electrocardiography, may also provide profound insights.

## Supplementary Material

kqae009_suppl_Supplementary_Tables
