# Supplementary material for: Impacts of workplace verbal aggression classified via text mining on workers’ mental health
Source: Occup Med (Lond). 2024 Feb 12;74(2):186–92. doi: 10.1093/occmed/kqae009 (PMC10990467; doi:10.1093/occmed/kqae009)
Supplement: kqae009_suppl_Supplementary_Tables [file kqae009_suppl_supplementary_tables.docx]

Supplemental Table 1 Regex terms used to detect each aggression types.

| Aggression types | Regex term |
| --- | --- |
| Attack on one’s appearance and personality | "/ブス/\|/はげ/\|/ハゲ/\|/性格/\|/カス/\|/キモイ/\|/クソ/" |
| Threats on life | "/死ぬ\|/死ねる/\|/殺す/" |
| Criticizing one’s job performance | "/バカ/\|/ばか/\|/馬鹿/\|/あほ/\|/アホ/\|/阿呆/\|/遅い/\|/頭.*/悪い/\|/役立つ/\|/使える/\|/ダメ/\|/駄目/\|/出来る/\|/能無し/\|/無能/\|/価値.*無/\|/ミス/\|/覚える/\|/ノロマ/\|/辞める/\|/やめる/\|/クビ/\|/首/\|/辞表/\|/退社/" |

Supplemental Table 2 Perpetrator of the aggressive language

| Perpetrator | n (%) |
| --- | --- |
| Superior | 63 (13%) |
| Coworker | 48 (10%) |
| Subordinate | 19 (4%) |
| Customer | 32 (6%) |
| Other | 13 (3%) |
| n = 500; There were cases in which a single participant reported multiple perpetrators. | |
